# The Use of Laser Sensing for Solving Meteorological Problems Related to Researching and Ensuring the Safety of Space Flights

**DOI:** 10.3390/s24216818

**Published:** 2024-10-23

**Authors:** Anatoly S. Boreysho, Maxim A. Konyaev, Sergey Y. Strakhov, Andrey V. Trilis, Natalia V. Sotnikova

**Affiliations:** Department of Laser Technology, Department of Radioelectronic Control Systems, Baltic State Technical University “VOENMEH”, St. 1st Krasnoarmeyskaya, 1, St. Petersburg 190005, Russia; boreisho_as@voenmeh.ru (A.S.B.); koniaev_ma@voenmeh.ru (M.A.K.); strakhov_siu@voenmeh.ru (S.Y.S.); trilis_av@voenmeh.ru (A.V.T.)

**Keywords:** space flight safety, meteorological support of the cosmodrome, laser remote sensing, Doppler lidar, heterodyne reception, wind shear

## Abstract

This paper is devoted to the issue of using laser (lidar) sensing to determine wind speed and direction when solving practical problems during the analysis of meteorological conditions in the area around spaceports. This issue is relevant both for making decisions on the possibility of a safe launch of a launch vehicle and for conducting search and rescue operations using groups of manned and unmanned aerial vehicles. Based on numerical and experimental modeling, it is shown that lidars provide highly accurate measurements of wind speed profiles and allow for the determination of weak wind shear in vertical and horizontal directions. This paper proposes a method for determining the main parameters of lidar sensing (range, resolution, detectability, etc.), which allows for the capabilities of this technology in solving the practical problems of meteorological monitoring to be predicted. Of particular interest in this article are experimental modeling data verifying the proposed calculation methods and the experimental determination of the capabilities of lidar diagnostics. This paper summarizes the data from multi-month experiments measuring wind speed in clear weather conditions when other means of remote diagnostics are ineffective. As a result of the experiments, a statistical distribution of the maximum range of wind speed measurement in normal weather conditions with natural variation in the concentration of scattering particles in the atmosphere was obtained. This article also discusses the possibility of combining lidars and meteorological radars for the meteorological support of flights.

## 1. Introduction

Laser remote sensing is currently one of the most effective tools for solving a whole range of scientific and practical problems, including those related to the safety of space flights [1,2] and the docking of spacecraft [3], the safety of aviation navigation [4], and meteorological and navigation support for flights of unmanned aerial vehicles (UAVs) and small space systems [5,6,7]. Laser remote sensing should be understood as a location-based study of remote objects or environments based on the analysis of the characteristics of laser radiation reflected (scattered) from them. The main practical problems solved with the help of laser remote sensing in the field of space flight safety include the following.

### 1.1. The Study of Meteorological Conditions in the Cosmodrome Area

It is the meteorological conditions that determine [8] the admissibility of launching a space rocket; the parameters of dispersion for the specific parts of a launch vehicle; the conditions for the use of high-precision radio–technical, radio–electronic, and optical–electronic systems as part of the technical launch complex; the measurement of complexes; and the specifics of the use of systems for refueling a launch vehicle and spacecraft with cryogenic components of rocket fuel and compressed gasses.

Meteorological conditions largely determine the possibility and effectiveness of using aviation and technical means to create special arrangements, such as air search groups, including those based on unmanned aerial vehicles; ground search groups; emergency rescue groups; and bases in the areas of impact of the separate parts of a launch vehicle.

In order to minimize the negative consequences of the impact of meteorological factors on the rocket, space equipment, and the personnel of the cosmodrome by means of continuously monitoring, analyzing, and forecasting the meteorological situation, and issuing the necessary recommendations to allow State Commissions and technical management to make decisions, meteorological support has been organized at the cosmodrome, one of the components of which is lidar systems for remote sensing of the atmosphere.

### 1.2. Conducting Search and Rescue Operations to Detect Landing Modules of Spacecraft or the Seperate Parts of a Launch Vehicle

This task can be effectively accomplished using a swarm (group) of unmanned aerial vehicles. A large volume of research in the past decade and a half has been conducted with the aim of developing methods and algorithms for controlling the coordinated movement of various types of autonomous (robotic) and, in particular, unmanned aerial vehicles. One of the important tasks in group control is the task of moving a group of unmanned aerial vehicles in formation in the form of a certain fixed geometric shape or in the form of a certain compact group (swarm) [9,10].

The movement of unmanned aerial vehicles in the form of a formation/swarm is used in a large number of practical problems, such as group search and monitoring, the creation of distributed phased antenna systems, the study of the composition of the atmosphere, etc. The problem of group control is particularly relevant for unmanned aircraft of the airplane type, which do not have a maneuverability as high as that of quadcopters and autonomous helicopters but surpass them in tasks that require a long flight duration. In addition, their advantages are their high load capacity, altitude movement, and speed. An effective solution to group control problems involves the correct consideration of and compensation for external influences, the most significant of which are wind effects. The influence of wind on the flight of any aircraft is a very significant factor that actively manifests itself both during a steady flight and particularly during the takeoff and landing stages, when the predictability of the aircraft trajectory is especially important [11,12].

### 1.3. Using Laser Remote Sensing Systems to Solve the Complex Navigation Problems of Landing Spacecraft on Unprepared Surfaces

The NASA Artemis program is particularly well known, and involves the development of technologies for landing a spacecraft on the lunar surface, which is being carried out by three companies: Blue Origin, Space X, and Dynetics.

For high-precision navigation during landing, a “machine vision” system is being developed, the key components of which are laser sensors (lidars). Lidars in combination with multi-range cameras and an intelligent data processing system will allow us to anticipate navigation hazards and adjust the course to navigate to a safe place. This technology was developed within the framework of the Safe and Precise Landing—Integrated Capabilities Evolution (SPLICE) project [13]. This approach to solving the problem of navigation during landing on the surface of a cosmic body has proven its effectiveness in the Chang’e-6 mission [14].

The basis of this technology is a coherent Doppler lidar, which facilitates the measurement of the range and speed of the landing module relative to the landing site [15,16,17].

This paper examines the capabilities of laser sensing technologies for solving the problems of providing meteorological support to enhance the safety of space flights.

## 2. Formulation of the Problem

Changes in the takeoff and landing characteristics of aircraft, including launch vehicles and UAVs, under the influence of a sharp weakening or strengthening of the wind have, in a number of cases, been one of the main causes of flight accidents. Wind shear is one of the most dangerous weather phenomena, significantly affecting the flight characteristics during takeoff and landing. Moreover, the wind characteristics which are critical for launches over the territories of the Baikonur and Vostochny Cosmodromes are observed in the layers of 0.5–2 km and 7–14 km [7]. Months with a high frequency of maximum wind shears over Baikonur include December and January, and over Vostochny these are February and November.

Wind shear is a vector difference in wind speeds at two points in space, relative to the distance between them. Wind shear constantly exists in nature and, for the most part, does not have a noticeable effect on flight dynamics. However, in some cases, very significant wind shear values are observed, which can affect aircraft of any class, which has been confirmed by the statistics for flight accidents [18]. For UAVs that can be used to conduct search and rescue operations in the landing area of the habitable module of the spacecraft or parts of the launch vehicle, wind shear is particularly critical due to their small size. In addition, when organizing the group control of a swarm of UAVs, wind shear can lead to random changes in its structure, and, in the worst case scenario, to a collision between UAVs. The intensity of wind shear is classified by the value of wind speed measurement on a certain spatial scale [19,20].

A sharp change in wind speed and/or direction is possible both in the horizontal direction (horizontal wind shear) and in the vertical direction (vertical wind shear). A vertical shear is a change in wind speed and/or direction with a change in flight altitude. Vertical wind shear, in turn, is usually divided into two types: positive and negative. A positive shear is a wind distribution when its speed at altitude is greater than at ground level. A negative shear is a wind distribution when the wind speed at altitude is less than at ground level. Wind shear in a certain atmospheric layer can be combined with turbulent and strong vertical air flows; therefore, significant wind shears are classified as hazardous external environmental influences. Unlike ice and thunderstorms, which can be detected visually or by onboard technical means, wind shear is a phenomenon invisible to traditional means of diagnosing meteorological conditions and is often sudden. The ICAO form recommends the following classification of wind shear magnitude [20]: weak—0–2 m/s at 30 m or 0–6.5 m/s at 100 m; moderate—2.0–4 m/s at 30 m or 6.5–13 m/s at 100 m; strong—4.0–6 m/s at 30 m or 13–20 m/s at 100 m; very strong—more than 6 m/s at 30 m or more than 20 m/s at 100 m

Thus, for the reliable detection of wind shear, it is necessary to have information on wind speed and direction with a spatial resolution of no more than 100 m.

Almost all major airports use Doppler weather radars, which facilitate the detection of a number of dangerous features of the wind field. However, radars that use the phenomenon of the backscattering of electromagnetic radiation on atmospheric aerosols in the radio range require a high aerosol density, and, therefore, are not always capable of detecting the dangerous features of the wind field and measuring the parameters of the vortex traces of aircraft in a “clean” atmosphere [21]. Unlike radars, a Doppler lidar probes the atmosphere with a narrow-beam laser beam in the optical range with high spatial and temporal coherence. The scattering of laser radiation occurs on aerosol particles that are always present in the atmosphere, which allows for working in a very clean atmosphere and achieving a data availability of about 100%.

## 3. Modeling of Doppler Lidar

The efficiency of a Doppler lidar is determined by several factors, some of which are related to the atmosphere, particularly the backscattering coefficient β [m^−1^sr^−1^], the attenuation coefficient α [m^−1^], and the structural constant of the refractive index. The hardware characteristics of the lidar, such as the diameter of the receiving telescope, the output energy of the laser, the bandwidth of the receiver, and the optical losses from the system, affect the operation of the lidar, but can be optimized taking into account the requirements of the parameters of the intended use. The coefficients β and α determine the power of the scattered probing radiation, which is collected by the receiving aperture of the lidar. Assuming single scattering, the signal from the single-frequency laser coming from the aerosol atmosphere at a wavelength of λ is described by the laser ranging equation [22], which is valid under conditions of a weak contribution of multiple scattering:

$$P_{r}\left(\lambda ,r\right)={\;\eta }_{\mathrm{all}}\eta_{g}\left(r\right)P_{0}\left[\frac{c\tau }{2}\right]\frac{A_{r}}{r^{2}}\beta \left(\lambda ,r\right)\exp\left\{-2\overset{r}{\underset{0}{\int }}\alpha \left(\lambda ,r\right)\mathrm{dr}\right\}+{\;P}_{\mathrm{brp}}$$

where P_0_—peak laser pulse power; r—range from which a signal is received; λ—laser wavelength; η_all_—overall efficiency of the lidar system; c—speed of light; τ—laser pulse duration; η_g_(r)—geometric factor (depends on the geometry of the lidar optical system; the maximum value is—1); A_r_—receiving antenna area; β(λ;r)—aerosol backscatter coefficient; α(λ;r)—aerosol attenuation coefficient; P_brp_—background radiation power.

The overall efficiency of the system η_all_ includes the efficiency of the transmitting optical system, the optical receiving system, the losses associated with the receiver alignment, the efficiency of the photodetector and other factors specific to each particular system. The geometric factor η_g_(r) describes the function of overlapping the fields of view of the receiving system and the laser spot. The dependence of the geometric factor on the distance is determined by the design of the system, particularly the divergence of the laser beam, the field of view of the receiving system, ans the linear distance and the angle of misalignment (disalignment) between the axes of the transmitting and receiving systems. The values of the geometric factor are dimensionless and lie in the range from 0 to 1.

The signal-to-noise ratio of the lidar signal can be written as follows:

$$SNR\left(r\right)=\frac{P\left(r\right)}{P_{noise}}=\frac{\langle i_{s}^{2}\rangle }{\langle i_{noise}^{2}\rangle }$$

where P(r)—signal radiation power from a distance r; P_noise_— noise power; and <i_s_^2^> and <$i_{noise}^{2}$> are the root mean square currents of the signal and noise, which in the case of heterodyne reception [23] are determined using the following formulas:

$$\begin{array}{c} \langle i_{s}^{2}\rangle =2\eta S^{2}P_{h}P_{s}\\ \langle i_{noise}^{2}\rangle =[2eSP_{h}+{\left(S*NEP\right)}^{2}+2eSP_{brp}]B \end{array}$$

where η—overall heterodyne detection efficiency; S—receiver Watt–ampere sensitivity; e—electron charge; P_h_—heterodyne power; NEP—receiver noise parameter; P_brp_—background radiation power; and B—bandwidth of the electronic path of the receiver’s amplification path.

In heterodyne detection, the power of the signal’s current P_s_ depends on the product of the signal power and the power of the heterodyne P_h_; therefore, the power of the heterodyne is selected in such a way that it is at maximum and satisfies two conditions, with the first being

$$2eSP_{h}\gg {\left(S*NEP\right)}^{2}+2eSP_{brp}$$

The condition of a significant excess in the local heterodyne radiation power over all other noises ensures that the detection mode is limited by shot noise. The second condition is to ensure the linearity and location of the signal in the dynamic range of the receiver amplification path [23]. For modern photodetectors operating in the IR range, this power is about 1 mW.

**Figure 1:**
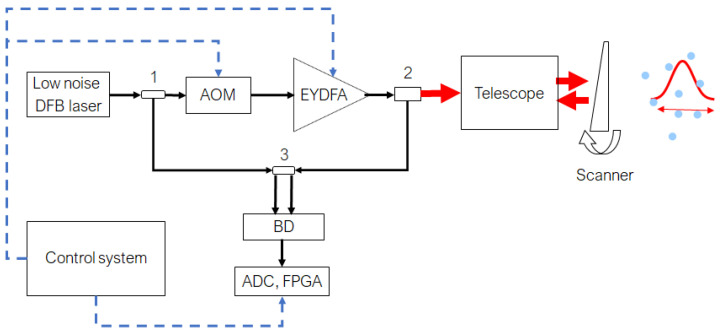
Block diagram of the coherent Doppler lidar. 1—fiber splitter; AOM—acousto-optic modulator; EYDFA—erbium-ytterbium doped fiber amplifier; 2—optical circulator; 3—2 × 2 fiber coupler. Black solid lines—PM fiber; dashed blue lines—electrical connections; red solid—open optical paths.

A block diagram of the coherent Doppler lidar is shown in Figure 1. Continuous wave (CW) radiation from low noise narrow linewidth DFB laser is divided into two parts, one part is used as a local heterodyne, the other is directed to the acousto-optic modulator (AOM). AOM can operate in two modes, continuous and pulsed. In continuous mode, it introduces only a frequency shift, in pulse mode, in addition to the frequency shift, the continuous input radiation is modulated by pulses with variable duration from 200 to 800 ns FWHM. This frequency-shifted CW or pulsed radiation are amplified by EYDFA, go through a transmit-receiving telescope, and are directed into the atmosphere by a scanner. Backscattered radiation collected by the transmit-receiving telescope and optical circulator separates outgoing and receiving beams. The incoming beam goes to a 2 × 2 fiber coupler, where it is mixed with the local oscillator. The balanced detector is used within the detection scheme to improve lidar sensitivity; after amplification, the heterodyne signal is sampled by means of a high-speed ADC with a frequency of 320 MS/s. The field programmable gate array (FPGA) processing board utilizes real-time Fast Fourier Transform to obtain the Doppler spectra. The control system sets the operating modes of the acousto-optic modulator, EYDFA, and the parameters for data processing on the FPGA. All optical paths in the lidar are based on a polarization-maintaining (PM) fiber.

The signal processing algorithm in a Doppler lidar consists of several main stages, the parameters of which vary depending on its type. The first stage consists of digitalizing the signal at the required frequency, then dividing the signal into data windows of a certain duration (RG), normalizing the data with a function (W), and calculating the Fourier spectrum (F) for the resulting array.

$$XR_{l}=\frac{1}{K}\overset{K}{\underset{i=1}{\sum }}F\left(W*RG\right)$$

**Figure 2:**
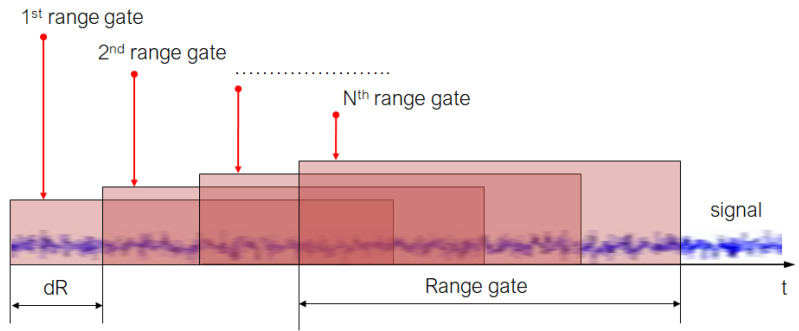
Signal range gating in Doppler lidar.

A graphical representation of the algorithm is shown in Figure 2.

In each spatial window, the Fourier spectrum is calculated and the parameters of the Doppler signal, frequency, spectrum width, and signal-to-noise ratio are determined.

Wind speed is calculated using the following formula:

$$\nu_{i}=\frac{\left(\frac{N}{2}-i\right)F_{ADC}\lambda_{Laser}}{4N}$$

where N—number Fourier spectrum frequency bins; F_ADC_—digitizing frequency; λ_Laser_—laser wavelength.

**Figure 3:**
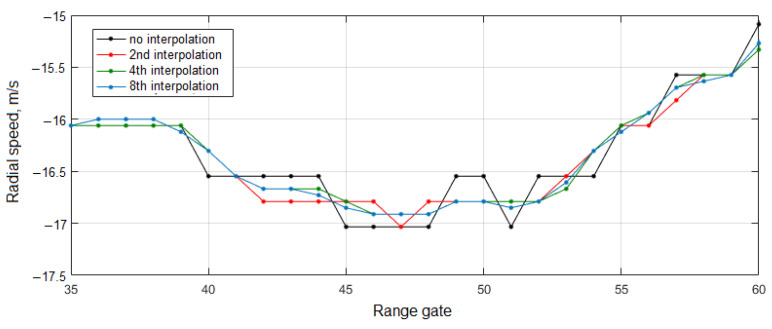
Radial velocity graphs with different orders of interpolation.

Since the calculated wind speed is a discrete function, it is necessary to use additional methods to improve the calculation accuracy. One such method is the interpolation of the obtained Doppler peak spectrum data using a polynomial function. Figure 3 shows graphs of the calculated radial velocity using the interpolation of polynomials of different orders.

**Figure 4:**
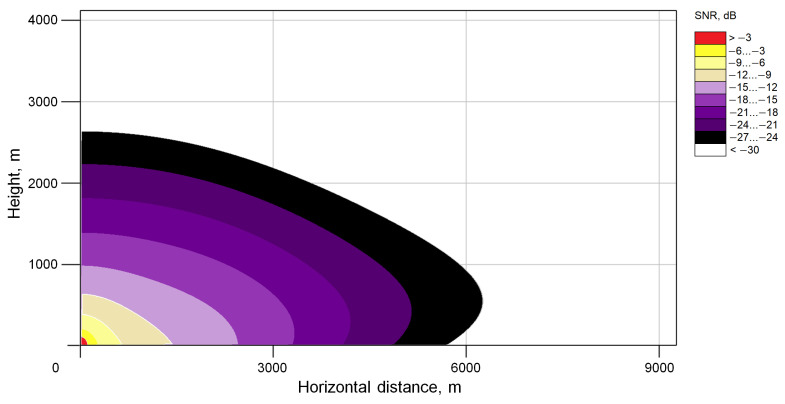
Signal-to-noise ratio distribution when scanning the upper hemisphere with a Doppler lidar.

The result of the calculation according to the model described above, for lidars with technical parameters similar to the WINDEX 5000 lidar (Table 1) and atmospheric parameters corresponding to a meteorological visibility range of 20 km, is shown in Figure 4, in which the SNR values when scanning the upper hemisphere are shown in different colors. The data are limited to the value of −30 dB, since smaller values do not allow for a reliable determination of the wind speed. The WINDEX 5000 lidar was manufactured by JSC “Laser Systems”, St. Petersburg, Russia; among the hardware developers are the authors of this article.

An analysis of the simulation results shows that wind speed detection is possible up to 2500 m, and up to 6000 for a horizontal path.

To reconstruct the full wind speed vector, measurements of three velocity projections are required. There are two modes which are commonly used for measuring the vertical profile of the full wind speed vector: VAD (Velocity Azimuth Display) and DBS (Doppler Beam Swinging). Both methods involve conical scanning with a laser beam; the difference lies in the number of measurement points: four in the DBS mode and continuous scanning in the VAD mode.

**Figure 5:**
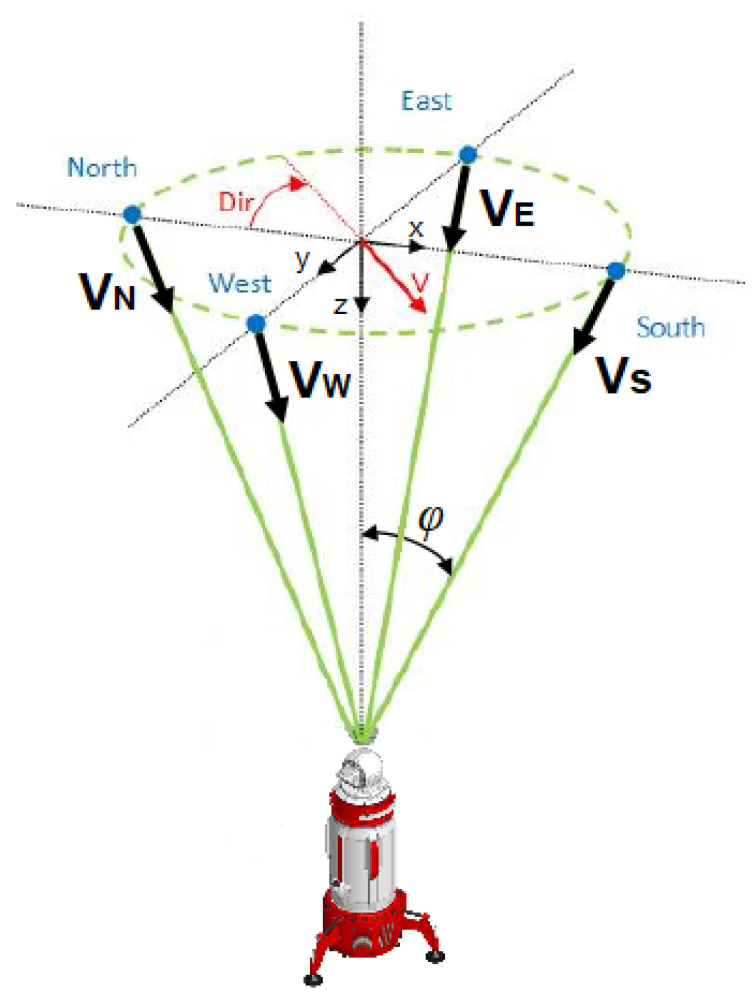
Scheme of measuring wind speed components with reference to the cardinal directions.

When reconstructing the full speed vector, the assumption that in the horizontal section of the cone the speed vector is uniform at all points limited by the cone generators is used. Although at least three projections are required to reconstruct the speed vector, the most informative and convenient scheme is the measurement scheme using four directions with reference to the cardinal directions, as shown in Figure 5. In this case, the relationship of the measured values with the speed components can be expressed as

$$\left\{\begin{array}{c} V_{N}=V_{x}\sin\varphi +V_{z}\cos\varphi \\ V_{S}=-V_{x}\sin\varphi +V_{z}\cos\varphi \\ V_{E}=V_{y}\sin\varphi +V_{z}\cos\varphi \\ V_{W}=-V_{y}\sin\varphi +V_{z}\cos\varphi \end{array}\right.$$

The reconstructed components of the velocity vector are defined as:

$$\left\{\begin{array}{c} V_{x}=\frac{V_{N}-V_{S}}{2\sin\varphi }\\ V_{y}=\frac{V_{E}-V_{W}}{2\sin\varphi }\\ V_{z}=\frac{V_{N}+V_{S}+V_{E}+V_{W}}{4\cos\varphi } \end{array}\right.$$

It is worth noting that, in the given scheme, it is possible to add a fifth direction vertically upwards for a precise measurement of the vertical component of the wind speed vector. However, such a modification of the DBS method is possible with the two-mirror design of the lidar scanner.

## 4. Results and Discussion

Currently, several wind lidars have been developed and are being produced, which are positioned as serial models, and among them the following can be distinguished: Windcube manufactured by Leosphere (France), Zephir manufactured by Qinetiq (England), Galion manufactured by SgurrEnergy (England), and WindTracer by the American company CTI Lockheed Martin [24].

**Figure 6:**
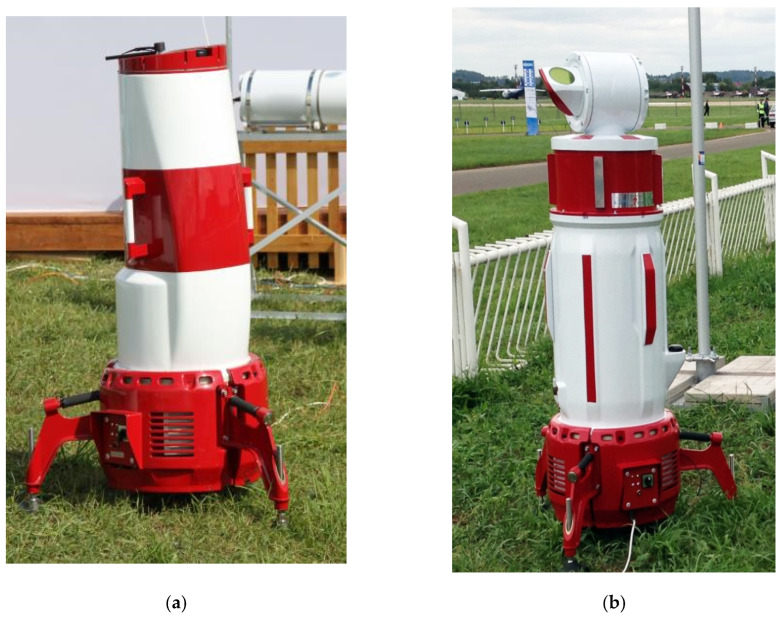
Appearance of (**a**) WINDEX 300 and (**b**) WINDEX 5000 lidars.

Over the past few years, JSC “Laser Systems”, with the participation of employees of the Laser Engineering Department of the D.F. Ustinov BSTU VOENMEKH, has developed a series of coherent Doppler lidars called WINDEX, which are based on semiconductor lasers and fiber components with the ability to reconstruct the wind speed profile [24,25]. The main technical characteristics of the WINDEX 300 (CW Doppler lidar) and WINDEX 5000 (Pulsed Doppler lidar) Doppler lidars are presented in Table 1. The appearance of the lidars is shown in Figure 6.

An important criterion for the efficiency of lidar systems is the wind speed detection range, which strongly depends on the optical properties of the atmosphere, particularly on the concentration of scattering particles. The distribution of scattering particles by height varies greatly depending on the time of day, time of year, type of underlying surface, and other factors that cannot be mathematically modeled. Therefore, the potential of wind lidars is estimated using the horizontal range of wind speed measurement.

To experimentally determine the maximum horizontal range of the wind speed measurements of the WINDEX 5000 lidar, multi-month wind speed measurements were performed for cloudless weather in the St. Petersburg area. As a result of these studies, a statistical distribution of the maximum range of wind speed measurements in normal weather conditions with natural variation in the concentration of scattering particles in the atmosphere was obtained.

**Figure 7:**
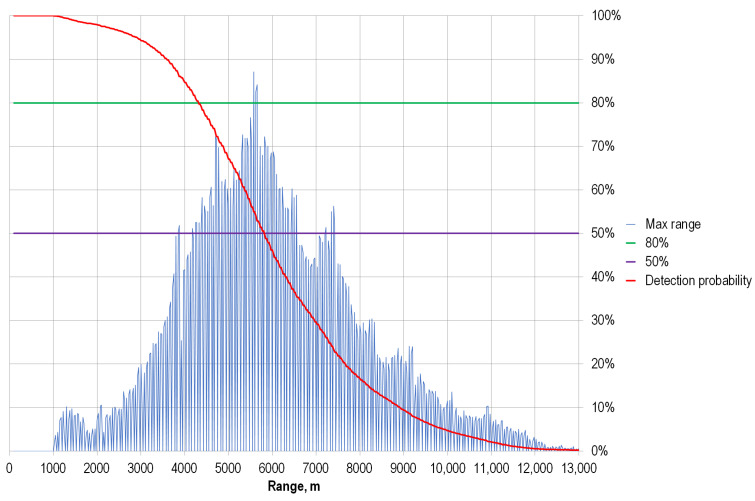
Maximum horizontal wind speed measurement range using WINDEX 5000 lidar.

The experimental data obtained are presented in Figure 7. The measurements were carried out in the horizontal direction with an elevation angle of 3 degrees. From the experimental data (Figure 4) it is evident that, depending on the state of the atmosphere, a wind speed measurement range of more than 4500 m is observed in 80% of cases; in 50% of cases, the wind speed is measured at distances of more than 5500 m, and the measurement distance is more than 8500 m in 10% of measurements. With a favorable distribution of scattering particles in the atmosphere, the wind speed measurement range can reach 12 km.

**Figure 8:**
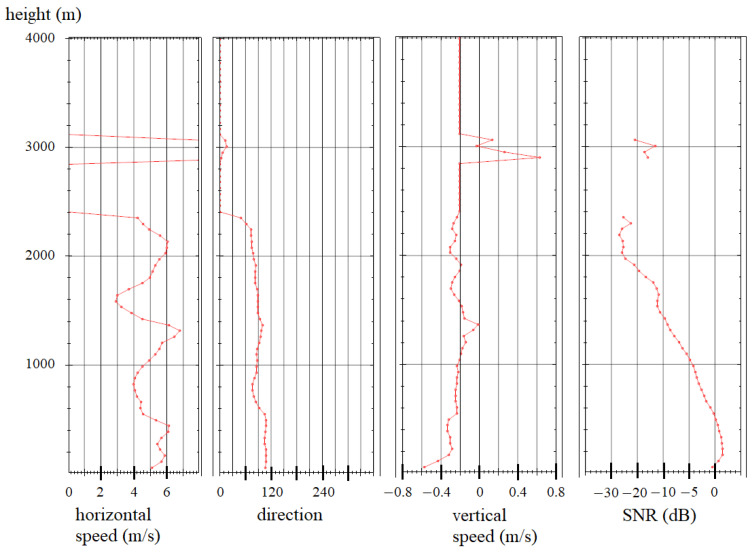
Wind speed altitude profile.

**Figure 9:**
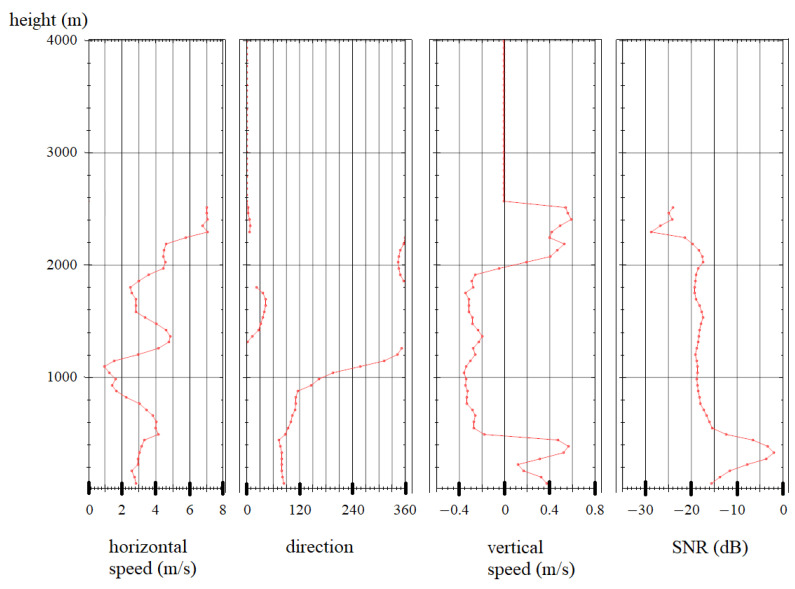
Measured wind speed altitude profile with class 1 wind shear.

Measuring the wind speed according to the scheme presented in Figure 5 makes it possible to obtain the wind speed distribution by height in real time and calculate the wind shear parameters caused by mixing of the atmospheric layers. Figure 8 and Figure 9 show typical graphs of the wind speed distribution by height. The minimum measurement height is determined by the pulse duration and is about 60 m. The averaging time for a single direction is 1 s.

The wind profile presented in Figure 8 shows a horizontal wind shear at an altitude of about 1400 m. The horizontal component of the speed vector changes; however, the vertical component and direction remain unchanged. Figure 9 shows a situation in which a change in the horizontal component of the wind and its direction is observed at an altitude of 1100–1300 m. The speed change was 4 m/s, and the direction changed by almost 270°, the vertical scale of changes is about 200 m. These indicators correspond to a moderate wind shear according to the ICAO form. It is worth noting the sharp changes in the vertical component of the wind at altitudes of 400 and 2000 m; the spatial scale of these changes is about 60 m.

**Figure 10:**
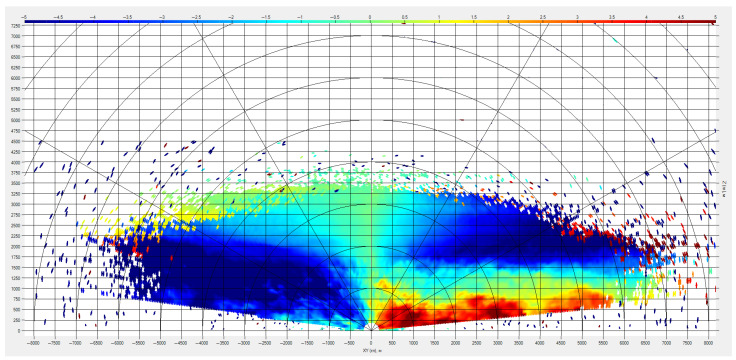
Vertical wind shear.

Wind shear can also be detected in the PPI (Plan Position Indicator—circular scanning at a low elevation angle) and RHI (Range Height Indicator—scanning in the vertical plane with a fixed azimuth angle) scanning modes. Figure 10 shows the distribution of radial wind speed when scanning in the RHI mode. The value of the radial speed and its sign are shown using color gradation. At an altitude of about 1500 m, a change in the direction and value of the speed in the vertical direction is observed. It is evident that the speed gradient is 8–10 m/s; such a difference on a scale of 500 m corresponds to a moderate wind shear according to the ICAO form.

Thus, laser sensing of the atmosphere using coherent Doppler lidars allows the distribution of wind speed and direction to be measured in the entire upper hemisphere, ensuring the detection of dangerous wind phenomena, such as wind shear of various types and intensities. Information on wind shear is an important element of ensuring safety during the flight, takeoff, and landing of various aircraft, airplanes, rockets, unmanned spacecraft, and small UAVs, as well as a necessary means for refining the effects of disturbances in the development and adjustment of UAV group control algorithms.

In conclusion, it should be noted that none of the currently existing meteorological systems can be considered to be universal or to solve the entire range of meteorological problems under various weather conditions and in various locations. One of the promising areas for improving meteorological systems operating in the conditions of the cosmodrome and providing information support for making decisions on the safety of the launch and flight of a launch vehicle is the integration of laser (lidar) and radar systems.

Indeed, for the operation of classical X-band radar systems, specific weather conditions are necessary; in particular, when the reflectivity of atmospheric objects is not lower than a certain value, which depends on the operating range, receiver sensitivity, noise level, etc. Therefore, measurements of wind speed, atmospheric turbulence, and detection of the wind shear effect in clear weather are impossible using radar [26,27].

At the same time, if we talk about an optical range radar (lidar) using a laser as a radiation source, then the opposite situation takes place. Since the wavelength of the radiation is about 1 μm, for its effective scattering in the atmosphere, the presence of an insignificant amount of aerosol is required, which takes place even in clear weather [26,28].

In [29], it is shown that each of the electromagnetic wave ranges applicable in a meteorological location has its own boundaries and optimum application in terms of the level of backscattering (“optical density” of meteorological formations), which can be determined from the physical processes of radiation passage through the atmosphere. In this case, it is possible to select a combination of probing ranges that will cover almost the entire scale of backscatter levels (all kinds of weather conditions) without losing the potential for determining meteorological parameters at long distances. One such combination could be a combination of X- and Ka-range radars with an IR lidar. Such a complex could become the basis for a meteorological support system for spaceports.

## 5. Conclusions

Lidar sensing of the atmosphere is one of the most effective tools for meteorological monitoring. The use of lidars is especially relevant to solving the practical problems of analyzing the meteorological situation in the area of spaceports both for deciding on the possibility of a safe launch of a launch vehicle and for conducting search and rescue operations using groups of manned and unmanned aerial vehicles. Lidars allow, with a high accuracy, in clear weather conditions, and when meteorological support equipment operating in the radio range is ineffective, the main atmospheric parameters that affect the safety of takeoff (and flight) to be measured: wind speed and direction, atmospheric turbulence, wind shear parameters, etc.

As a result of numerical modeling and experiments, it was shown that lidars provide wind speed profile measurements with an accuracy of no worse than 0.1 m/s, which allows weak wind shear to be determined in the vertical and horizontal directions. The distances achieved in the experiments are up to 5 km in the horizontal direction and up to 4 km in height. When changing the lidar parameters, the specified distances can be increased.

The paper proposes a method for calculating the main parameters of lidar sensing (range, resolution, detectability, etc.), which allows predicting the capabilities of this technology in solving practical problems of meteorological monitoring. The calculation method is based on solving the lidar equation, taking into account the parameters of the receiving and transmitting channel.

A method for constructing a wind speed profile based on processing lidar measurement data are proposed, and experimental studies are carried out that verify the proposed methods and demonstrate the capabilities of lidar diagnostics of the state of the atmosphere.

## Figures and Tables

**Table 1:** Modeled result of the direct problem solution.

Parameter	WINDEX 300	WINDEX 5000
Measurement ranges (horizontal)	from 5 to 300 m	from 60 to 5000 m
Speed measurement range	from 1 to 40 m/s	from 1 to 55 m/s
Wind direction measurement range	from 0 to 360°	from 0 to 360°
Laser wavelength	1550 nm	1550 nm
Laser pulse duration	continuous	400 ns
Energy of impulse	1 Watt	150 μJ
Spatial resolution	±10% of the focusing distance	40 m
Wind speed and direction data update time	4 s	1–10 s
Scan mode	VAD	VAD, DBS, RHI, PPI, LOS
Weight	70 kg	150 kg
Overall dimensions	450 × 900 × 1300 mm	885 × 1005 × 1745 mm

## Data Availability

Data are contained within the article.

## Nomenclatures

A_r_	receiving antenna area	η	overall heterodyne detection efficiency
c	speed of light	A(*λ*,*r*)	aerosol attenuation coefficient
e	electron charge	β(*λ*,*r*)	aerosol backscatter coefficient
B	bandwidth of the electronic path of the receiver’s amplification path	λ	laser wavelength
NEP	receiver noise parameter	η_all_	overall efficiency of the lidar system
P_h_	heterodyne power	τ	laser pulse duration
S	receiver watt-ampere sensitivity	η_g_(r)	geometric factor
P_brp_	background radiation power		
P_o_	peak laser pulse power		
r	range from which a signal is received		
P(r)	signal radiation power from a distance r		
P_noise_	noise power		
